# Health providers’ reasons for participating in abortion care: A scoping review

**DOI:** 10.1177/17455057241233124

**Published:** 2024-03-01

**Authors:** Bronwen Merner, Casey M Haining, Lindy Willmott, Julian Savulescu, Louise A Keogh

**Affiliations:** 1 Melbourne School of Population and Global Health, University of Melbourne, Parkville, VIC, Australia; 2 Australian Centre for Health Law Research, Faculty of Business and Law, Queensland University of Technology, Brisbane, QLD, Australia; 3 Oxford Uehiro Centre for Practical Ethics, University of Oxford, Oxford, UK; 4 Centre for Biomedical Ethics, Yong Loo Lin School of Medicine, National University of Singapore, Singapore; 5 Murdoch Children’s Research Institute, Parkville, VIC, Australia; 6 University of Melbourne, Parkville, VIC, Australia

**Keywords:** abortion, conscientious objection, midwives, nurses, obstetrics and gynaecology, physicians, termination of pregnancy

## Abstract

**Background:**

There is a global shortage of health providers in abortion care. Public discourse presents abortion providers as dangerous and greedy and links ‘conscience’ with refusal to participate. This may discourage provision. A scoping review of empirical evidence is needed to inform public perceptions of the reasons that health providers participate in abortion.

**Objective:**

The study aimed to identify what is known about health providers’ reasons for participating in abortion provision.

**Eligibility criteria:**

Studies were eligible if they included health providers’ reasons for participating in legal abortion provision. Only empirical studies were eligible for inclusion.

**Sources of evidence:**

We searched the following databases from January 2000 until January 2022: Medical Literature Analysis and Retrieval System Online, Excerpta Medica Database, Cumulative Index to Nursing and Allied Health Literature, ScienceDirect and Centre for Agricultural and Biosciences International Abstracts. Grey literature was also searched.

**Methods:**

Dual screening was conducted of both title/abstract and full-text articles. Health providers’ reasons for provision were extracted and grouped into preliminary categories based on the existing research. These categories were revised by all authors until they sufficiently reflected the extracted data.

**Results:**

From 3251 records retrieved, 68 studies were included. In descending order, reasons for participating in abortion were as follows: supporting women’s choices and advocating for women’s rights (76%); being professionally committed to participating in abortion (50%); aligning with personal, religious or moral values (39%); finding provision satisfying and important (33%); being influenced by workplace exposure or support (19%); responding to the community needs for abortion services (14%) and participating for practical and lifestyle reasons (8%).

**Conclusion:**

Abortion providers participated in abortion for a range of reasons. Reasons were mainly focused on supporting women’s choices and rights; providing professional health care; and providing services that aligned with the provider’s own personal, religious or moral values. The findings provided no evidence to support negative portrayals of abortion providers present in public discourse. Like conscientious objectors, abortion providers can also be motivated by conscience.

## Introduction

Abortion is a common, safe healthcare intervention which includes information provision, abortion management and post-abortion care.^1^ The provision of safe abortion, conducted consistently with clinical guidelines, is key to achieving the Sustainable Development Goals relating to gender equality, good health and well-being.^2^ However, a key barrier to safe abortion access is the shortage of providers.^3^ The deficit of skilled health providers is especially critical in countries which also have high levels of unsafe abortion and associated maternal deaths. In addition, most countries, including high-income ones, have shortages of providers in regional and remote areas, and have most providers concentrated in the private sector generating inequities.^3^ Exacerbating these issues are legal or policy barriers, including institutional objection, unwillingness of health providers to participate in abortions and community stigma.^1,4^

Negative portrayals of abortion providers in public discourse may be consistent with a stigmatized perception of abortion provision. In the United States, a study of abortion plotlines on television from 2005 to 2014 showed physicians offering legal abortion care in medical facilities were portrayed as compassionate and committed to their patients.^5^ However, the negative portrayal of illegal abortion care reinforced the stigma that abortion providers lacked concern for their patients’ safety and well-being. In Great Britain, a qualitative analysis of print media about abortion in 2010 showed negative framings of abortion providers predominated. Framings included the portrayal of abortion providers as neglecting their responsibility to inform patients about abortion risks. Advertisements for abortion services were also described as ‘sick’, ‘grotesque’ and ‘tragic’.^6^ A synthesis of empirical research is needed to inform the evidence base about whether such portrayals of abortion providers are accurate.

In the ‘legitimacy paradox’, Harris theorized why abortion providers may be represented as dangerous, deviant or illegitimate.^7^ She theorized that abortion stigma discouraged providers from discussing their work fuelling a perception that abortion provision was unusual and non-standard. This lack of discussion contributed to abortion work being seen as ‘deviant’ and not the type of work performed by legitimate, mainstream health providers. Yet, despite the challenge of stigma and its negative impacts, some health providers continue to participate in abortion provision. A scoping review identifying health providers’ reasons for participating in abortion may help to understand the characteristics of this group.

Another dominant discourse that may discourage potential providers links ‘conscience’ with refusal to participate in abortion.^8^ Conscience refers to a person’s set of core moral beliefs that are integral to their sense of identity.^9^ Although previous research has shown that some health practitioners feel a conscientious obligation to provide abortion, laws typically protect only those who conscientiously object.^10,11^ Similarly, research tends to focus on objectors, rather than the providers of abortion.^12–14^ Identifying whether conscientious provision is a reason for participation across the empirical literature will challenge the discourse linking conscience solely with refusal.

In a scoping review of conscientious objectors and other non-participating providers, refusal to participate was also influenced by individual characteristics, systems and clinical practice factors, professional ethos and emotional labour considerations (including fear of the emotional impact of participating in the procedure).^15^ The limited available evidence suggests that decisions to participate in abortion may be similarly complex. In a South African study of nurses, Potgieter and Andrews^16^ found that reasons for participation in abortion could be framed broadly within three main discourses: public health, rights and sociocultural. More recently, in a study from the United States, Czarnecki et al.^17^ concluded abortion participation decisions were influenced by a diversity of factors beyond personal beliefs, including work experiences, social and institutional contexts. A scoping review will assist in identifying the key reasons for participating in abortion care across a broad range of studies.

In this review, we aim to map the empirical evidence base for health providers’ reasons for participating in abortion provision, in settings where abortion is lawful. We have limited the review to lawful settings because providers’ reasons may be different when it is necessary to break the law to provide an abortion. To our knowledge, this is the first review on the topic of health providers’ reasons for participating in abortion provision.

## Methods

Our research question was: what is known from the existing empirical literature about the reasons that health providers participate in legal abortion provision?^18^ This scoping review was informed by the Joanna Briggs Institute (JBI) methodology.^19^ The Preferred Reporting Items for Systematic Reviews and Meta-Analyses (PRISMA) Extension for Scoping Reviews has also been used in reporting this review.^20^

### Selection criteria

The selection criteria are shown in Table 1. Consistent with a scoping review, we used a broad definition of health providers to enable us to include a wide range of studies. We defined health providers to include both clinical and non-clinical staff. Student health providers were only included as participants when they were combined with health providers in a study. The phenomenon of interest was the reason/s that health providers participated in legal abortion provision. We included quantitative, qualitative, and mixed methods study designs in our review.

**Table 1.** Selection criteria.

	Inclusion criteria	Exclusion criteria
Participants	• Health providers (both clinical and non-clinical staff) who participate, or intend to participate, directly or indirectly in legal abortion provision (including pre-abortion counselling and imaging, medical or surgical abortion, post-abortion care, and abortion service management or administration) in specialist and general healthcare settings • Student health providers will only be included as participants when they are combined with health providers in a study	• Health providers who offer abortion outside of the healthcare system and do not have formal qualifications • Health providers who are conscientious objectors (including partial objectors)
Concept	• Factors that motivate health providers to participate in legal abortion provision	• Health providers’ experiences and perceptions of abortion provision that do not relate to their reasons for participation • Education and training interventions (e.g. abortion training or Providers’ Share Workshops)
Context	• Countries where abortion provision is lawful (as stated in the published article)	• Countries where abortion provision is not lawful (as stated in the published article)

### Identifying relevant studies

The following databases were searched on 20 January 2022:

- Medical Literature Analysis and Retrieval System Online (MEDLINE)
- Excerpta Medica Database (Embase)
- Cumulative Index to Nursing and Allied Health Literature (CINAHL)
- ScienceDirect
- Centre for Agricultural and Biosciences (CAB) International Abstracts (including Global Health)

The search strategies are shown in Appendix 1 in the Supplemental Material. Searches were limited to English language studies only, due to the lack of translating capacity in the team and the complexity of translating qualitative studies. We also limited the search to include studies from 2000 onwards. This limitation was implemented to reflect the global trend of liberalization of abortion after 2000.^21^

Reference lists of included studies, and relevant systematic and literature reviews were also searched for eligible studies.

Sources of unpublished studies/ grey literature were searched using ProQuest Theses and Dissertations (using keywords such as ‘abortion providers’ and ‘motivations’). The websites of relevant government and non-government organizations, such as the World Health Organization, Guttmacher Institute and Marie Stopes International, were searched using each website’s internal search function. The first three pages of Google was also searched using the keywords ‘abortion providers motivations’.

### Study/source of evidence selection

All titles and abstracts were screened by two authors (B.M. and C.M.H.) independently using Covidence software.^22^ Potentially included abstracts were then retrieved in full text and screened by two authors (B.M. and C.M.H.) independently to determine if they met the eligibility criteria. Any discrepancies arising during the two screening stages were discussed by B.M. and C.M.H., and resolved by consensus.

### Data analysis

Data were extracted from the included studies by one reviewer (B.M.) using a data extraction tool developed and piloted with five studies by the reviewers. Extracted data included author, title, country, city, health service setting, aim of study, sampling and recruitment processes, number and type of participants, data collection and analysis methods, and reasons for participating in abortion care.

Preliminary categories of reasons were developed from the extracted data by B.M. in NVivo 12. These categories were initially informed by providers’ reasons identified in two studies whose aims most closely matched the aims of the review.^16,17^ These studies were used to form the initial framework because they were the richest in data relevant to the topic. The initial framework comprised the following categories from Potgeiter and Andrews:^16^ public health, rights and sociocultural. The article by Czarnecki et al.^17^ informed the development of subcategories within the ‘sociocultural’ category, specifically ‘professional, community and organizational contexts’. The categories were then revised with input from all authors (B.M., C.M.H., L.W., J.S. and L.A.K.) until they sufficiently reflected all extracted data. The studies were then charted across these categories. Most of the data were qualitative; however, relevant quantitative data were also charted to the relevant category.

Consistent with scoping review methods, studies were not quality-appraised and findings were not ‘weighted’ according to certainty and generalizability.^23^

### Protocol

The protocol for this review was registered on Open Science Framework on 13 January 2022.^24^ Changes made between the protocol and the review are shown in Appendix 2 in the Supplemental Material.

## Results

### Included studies

**Figure 1.**
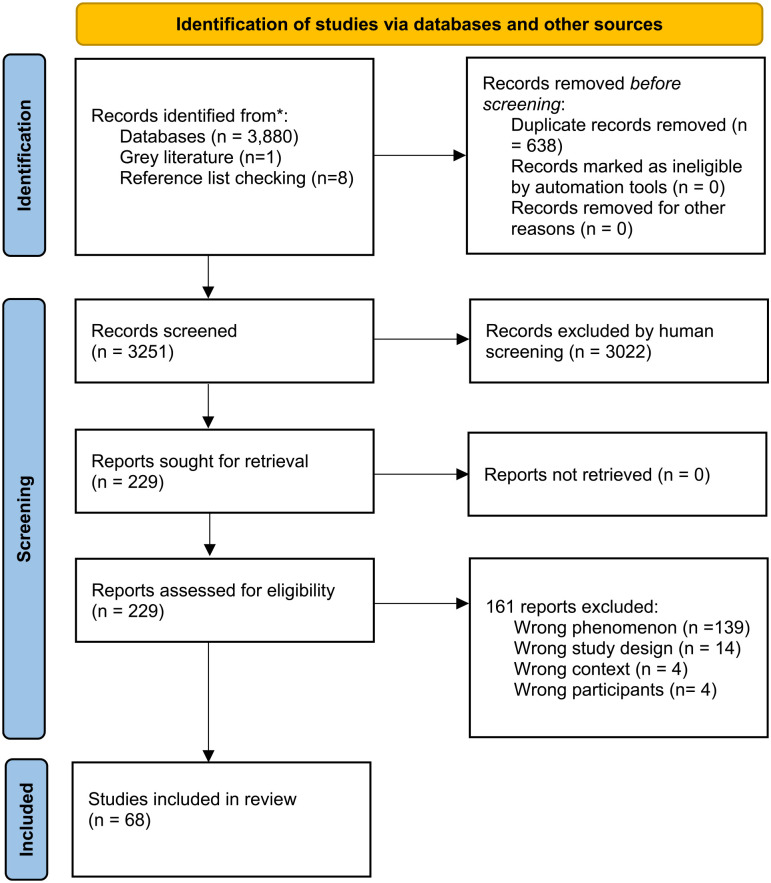
PRISMA diagram. *From*: Page et al.^25^

After duplicates were removed, B.M. and C.M.H. screened 3251 titles and abstracts and then assessed 229 full-text articles. After 161 articles were excluded, 68 studies met the inclusion criteria. The PRISMA diagram is shown in Figure 1.

The included studies were from North America (n = 28), Africa (n = 19), Europe (n = 11), South America (n = 3), Asia (n = 3) and Oceania (n = 3) with a further study involving multiple regions. Publication types included 62 peer-reviewed primary studies and 6 theses. Around 60 studies used qualitative methods, 7 used mixed methods and 1 study used quantitative methods.

Most of the included studies included clinical health providers as participants. However, 30 studies included doctors (including medical specialists), followed by nurses (n = 28), midwives (n = 20), other clinicians (e.g. pharmacist, social worker, sonographer; n = 20) and clinicians whose discipline was not stated (e.g. health professionals or abortion providers; n = 10). Eight studies included health providers in non-clinical roles (e.g. clinic manager, receptionist, volunteer).

The characteristics of included studies table are shown in Table 2.

**Table 2.** Characteristics of included studies.

Study ID/country	Aim	Study type	Recruitment methods	Selection criteria	Participants
Andaya and Campo-Engelstein^26^/US	Explores ‘how clinicians think about the capacity of periviable entities to feel pain and how these ideas shape clinical practice and understandings of compassionate care’	Qualitative, semi-structured interviews	Professional networks and snowball sampling	Physicians who provide second trimester abortion care and NICU clinicians	20 clinicians (11 physicians who provided abortion care in second trimester and 9 NICU clinicians)
Andersson et al.^27^/Sweden	Explores the perceptions and experiences of nurses and midwives involved in second trimester medical termination of pregnancy (TOP)	Qualitative, in-depth interviews	Invited to participate via workplace meetings and provision of verbal and written information about the study	Nurses/midwives with experience of abortion care at three gynaecological care units at a large hospital	17 nurses and 4 midwives
Aniteye and Mayhew^28^/Ghana	Explores the implementation of abortion law and policy	Qualitative, in-depth interviews	Not reported	Health practitioners with substantial knowledge and experience of abortion	43 health professionals (including 15 obstetricians, 14 midwives and 14 other health professionals)
Aniteye and Mayhew^29^/Ghana	Explores ‘the development of de jure law and policies on comprehensive abortion care in Ghana, the de facto interpretation and implementation of those policies, and assess what role globalization played in the transition in abortion care in Ghana’	Qualitative, in-depth interviews and document analysis	Health professional participants were identified by the Heads of reproductive and child health units in a range of public and private hospitals Recruitment of policymakers/NGOs not reported	Knowledge of, and interest in, abortion care	58 participants (including 15 obstetricians, 14 midwives, 12 other health professionals, 14 policymakers, 3 representatives of development agencies/NGOs)
Armour et al.^30^/New Zealand	Explores ‘the lived experience of midwives who provide care during termination of pregnancy (TOP) after 20 weeks’	Hermeneutic phenomenology, in-depth interviews	Poster invitations in tea rooms of maternity units	Midwives who provide TOP after 20 weeks	8 midwives
Baird^31^/Australia	Explores ‘the material and discursive negotiations through which some doctors facilitate the ongoing availability and indeed improvement of abortion services’	Qualitative, in-depth interviews	Not reported	Doctors who provided abortions	4 doctors
Baldiga^32^/USA	Explores how codification of restrictive state legislation affects US abortion workers Understand the mechanisms of positive identity work used by abortion workers	Qualitative, in-depth interviews	Cold-calling abortion activists and abortion clinics plus snowball sampling	Current and former abortion workers who live and work in states with restrictive abortion legislation	11 participants (including 8 physicians, 2 certified nursing assistants, 1 clinic manager, 1 office manager)
Becker and Hann^33^/US	Explores ‘the meaning-making processes during post-abortion tissue viewing through a focus on the providers who facilitate this practice’	Qualitative, symbolic interactionism, semi-structured interviews	Emails sent to independent clinics Clinics that agreed to participate then sent individual email invitations to their staff	Abortion providers from independent clinics. Abortion providers were defined as ‘any staff member whose duties included patient care and interaction with post-abortion tissue’	25 abortion providers
Bishop^34^/Canada	Explores ‘nurses’ experiences of caring for women having second trimester pregnancy terminations for fetal anomalies through labour induction techniques’	Qualitative, phenomenology, in-depth interviews	Invitation to participate via staff meetings at sampled hospitals plus flyers left in staff areas	Nurses who: (a) worked in the units of three Ontario hospitals providing second trimester abortion for foetal anomalies (b) provided care for these women. Nurses who provided only pre- and post-abortion genetic counselling were excluded	11 nurses
Britton et al.^35^/USA	Explores how abortion providers perceive their professional identity, and how adherence with restrictive laws influence that professional identity	Secondary analysis of interview data	List of abortion facilities in North Carolina compiled through accessing National Abortion Federation database, Google searches, and professional networks Invitation letters sent to facilities, with follow-up phone calls and emails	Professional abortion provider experience in North Carolina following the introduction of Women’s Right to Know (WRTK) legislation and experience in North Carolina prior to WRTK, or in others states with less restrictive legislation	31 participants across 11 sites (including 17 doctors, 9 registered nurses, 1 physician assistant, 1 counsellor, 3 clinic administrators)
Cardenas et al.^36^/Uruguay	Explores perceptions of abortion clients and health providers 2 years following decriminalization and how abortion stigma manifests among these individuals	Qualitative, in-depth interviews	For health providers: Invitation for interview followed participation in a quantitative study.	Abortion providers	10 abortion providers including physicians, midwives, social workers, and a psychiatrist.
Chiapetta-Swanson^37^/Canada	Explores nurses’ experiences of genetic termination work	Qualitative, in-depth interviews	Presented about research at staff meetings	Nurses who managed the genetic termination for foetal anomaly in four Canadian hospitals	41 nurses (including 35 clinical care nurses, 3 nursing directors, 3 were clinical nurse specialists)
Chowdhary et al.^38^/US	Explores ‘the consequences of restrictive environments for the abortion workforce’	Qualitative, in-depth interviews	Presented about research at national meetings of abortion providers	Physicians who were credentialed and provided abortions in at least one southern US state in either a hospital or clinic setting	12 physician abortion providers
Cignacco^39^/Switzerland	Explores midwives’ experiences of abortion for foetal abnormalities	Qualitative, in-depth interviews	Invited in writing and word of mouth	Midwives working in a delivery unit of a hospital	13 midwives
Cleeve et al.^40^/Uganda	Explores midwives’ views on post-abortion care (PAC) to improve the understanding of the quality of care	Qualitative, in-depth interviews	Written invitation to participate	Midwives working at the emergency gynaecological ward in national referral hospital where PAC is provided	22 midwives
Crookston^41^/USA	Explores ‘the experiences of non-physician employees and volunteers at an independently owned abortion clinic in the Midwestern United States’	Qualitative, in-depth interviews	Not reported	Non-physician employees and volunteers at a ‘particularly vulnerable, independently owned abortion clinic assailed by state-mandated restrictions and TRAP laws’	11 non-physician employees and volunteers
Czarnecki et al.^17^/USA	Explores ‘the diverse factors and experiences that healthcare workers shared in their accounts of working in a hospital that provides abortion services and how these factors shaped their decisions about involvement in abortion care’	Qualitative, in-depth interviews	Flyers in staff areas, emails to group lists	‘We recruited participants with different professional roles and different levels of power (in this case, doctors and nurses); at different stages of professional development (attending physicians and residents); responsible for different tasks in abortion work (administering anesthesia, performing surgical procedures, ordering medications for labor induction abortions, and carrying out those orders or performing other aspects of nursing care)’ in the Labour and Delivery unit of a large Midwestern teaching hospital	50 healthcare professionals (including 19 nurses, 15 anaesthetists, 6 maternal–foetal medical specialists, 10 obstetrics and gynaecology residents)
Dawson et al.^42^/Australia	Explores provision of and referral for medical abortion (MA) by general practitioners (GPs)	Qualitative, in-depth interviews and focus group	Invitation letters sent to sampled GP practices Study also advertised in newsletters of local health districts plus snowball sampling	GPs in eight areas of metropolitan, regional and remote NSW (sampled to reflect community and general practice diversity)	32 GPs including 8 MA providers (24 did not provide MAs)
Dobie et al.^43^/US	Explores ‘reproductive health care in rural Washington State, reasons given by providers for not offering abortions and providers’ willingness to use medical abortifacients’	Quantitative, written survey	Provider lists generated from professional and state licencing databases	Licenced rural Washington State abortion providers (including medical, midwifery, physician assistants and nurse practitioners)	707 survey respondents (including physicians (67.5%), certified nurse midwives (2.1%), nurse practitioners (14.9%), physician assistants (14.4%) and licenced midwives (1.1%))
Dowler et al.^44^/Canada	Explores midwives’ attitudes towards expanding their scope of practice to include MA in British Colombia	Qualitative, semi-structured interviews	Invited to participate via email from professional association	British Colombia registered midwives	15 midwives
Engelbrecht et al.^45^/South Africa	Explores ‘the identification of problems, constraints and impediments encountered in the process of implementing the Choice on Termination of Pregnancy Act in the Free State’	In-depth, unstructured interviews and mixed methods survey	Women: Eligible participants were alerted to the study by the nurse/social worker who conducted the pre-abortion counselling session. The details of women willing to participate were forwarded to researchers Abortion providers: TOP facility manager circulated surveys to the staff Referring clinicians: Contact details provided to researchers by TOP facility managers	Women who had experienced an abortion Health professionals and social workers who provide abortion services Health professionals and social workers who are able to refer to abortion services	75 women who had experienced an abortion 16 health professionals and social workers who provide abortion services 63 health professionals and social workers who are able to refer to abortion services
Ewentu et al.^46^/Ethiopia	Examines how abortion providers who have religious beliefs negotiate the conflicting demands of their jobs and their religious values	Qualitative, in-depth interviews	Cold-calling abortion services in either public hospitals or private abortion clinics in Addis Ababa	Healthcare professionals involved in abortion services in either private/non-governmental clinics or in public hospitals	30 abortion providers including nurses, midwives, public health specialists, general practitioners and OBGYNs 22 who provided abortions directly 8 who worked with abortion in an administrative capacity or provided contraceptives and post-abortion care services
Fernandez Vazquez and Brown^47^/Argentina	Describes ‘some key moments of change in the social and legal contexts, allowing providers in the public health system in the Metropolitan Area of Buenos Aires to go from invisibility to visibility, and from hiding their experiences to showing them with pride’	Qualitative, in-depth interviews	Recruited through convenience, previous contact and snowball sampling	Health professionals providing access to abortion in the public system	27 participants (including 16 general practitioners, 4 gynaecologists, 2 social workers, 2 psychologists, 1 sociologist, 1 paediatrician who attends counselling for adolescents, 1 pharmacist)
Footman et al.^48^/Zambia	Explores ‘the experiences and motivations of pharmacy workers who sell medication abortion (MA) drugs in Lusaka’	Qualitative, in-depth interviews	Recruited as part of a larger study of an intervention aiming to increase women’s access to information about medication abortion	Pharmacy workers responsible for selling MA drugs	16 pharmacy workers
Freeman and Coast^49^/Zambia	Explores ‘the experiences of practitioners who conscientiously object to abortion alongside those who do not to investigate divergences – or similarities’	Qualitative, in-depth interviews	Recruited through gatekeepers at local hospitals	All types of healthcare practitioners involved in women’s abortion clinical pathway	51 health practitioners (both objectors and non-objectors)
Gallagher et al.^50^/United Kingdom	Explores the views of nurses employed in abortion services	Qualitative, in-depth interviews	Invited to take part following an introduction to the study by the research team	Nurses employed in three registered abortion clinics (selected because they provide abortion up until the legal limit of 24 weeks) Involved in the provision of either medical or surgical abortion or both	9 nurses across three clinics
Garel et al.^51^/France	Explores midwives’ experiences performing abortions for foetal abnormality	Questionnaire, closed and open questions	Personally delivered invitations to all midwives present in select hospitals during the survey period	Midwives from 6 public maternity hospitals	87 midwives
Glenton et al.^52^/Multi-country (Bangladesh, Ethiopia, Nepal, South Africa and Uruguay)	Explores ‘factors influencing the implementation of role expansion strategies for non-physician providers to include the delivery of abortion care’	Case study synthesis with document analysis plus in-depth interviews	Invited key informants directly	Key informants from NGO and government and research institutions involved in implementing or assessing abortion care programmes in the five countries	12 key informants
Gmeiner et al.^53^/South Africa	Explores nurses’ experiences of direct involvement in TOP	Qualitative, phenomenology, interviews	Not reported	Nurses voluntarily directly involved with termination services	Nurses directly involved with termination services (number of participants not reported)
Hammarstedt et al.^54^/Sweden	Describes gynaecologists’ experiences working in abortion care	Questionnaire, both structured and semi-structured questions	Questionnaire sent to a random sample of 269 gynaecologists	Gynaecologists active in their professions	228 gynaecologists
Handa and Rosenberg^55^/Canada	Surveys ‘Ontario midwives’ general attitudes towards abortion and willingness to incorporate abortion into the midwifery scope of practice’	Cross-sectional survey, closed and open questions	Questionnaire sent to all registered midwives in Ontario (n = 523)	Registered midwives	359 midwives
Harries et al.^56^/South Africa	Explores ‘knowledge, attitudes and opinions of health service providers who are likely to play a critical role in determining access to and the quality of’ abortion services	Qualitative, in-depth interviews and focus group	Snowball sampling	‘Health care providers and health care managers who were working in facilities that provided abortion services in the public, private and NGO sectors’	38 participants including ‘some providers [who] provided abortions and some assisted with the procedure and provided pre- and post-abortion counselling. Others restricted their involvement to tasks solely relating to pre-abortion care, such as performing ultrasounds to determine gestational age and referral to a designated abortion facility’
Holcombe et al.^57^/Ethiopia	Explores ‘midwives’ attitudes towards abortion to understand their decisions about service provision’	Mixed methods, survey and in-depth interviews	Surveys fielded at Ethiopian Midwives Association (EMA) meeting. Unclear how midwifery students were recruited for interviews	218 midwives and midwifery students	188 surveys returned from midwives 12 interviews with third year midwifery students
Homaifar et al.^58^/USA	Explores ‘the motivations around and practices of abortion referral among women’s health providers’	Self-administered survey, with closed and open responses	Surveys sent to eligible clinicians identified via the Health Professions Tracking Service	Physicians, advanced-practice clinicians in family medicine and obstetrics–gynaecology (OBGYN) whose self-identified specialty was obstetrics/gynaecology, family medicine, women’s health or nurse midwifery	431 health practitioners (including physicians and advanced-practice clinicians)
Joffe and Yanow^59^/USA	Explores the impact of becoming an abortion provider on advanced practice clinicians’ professional identities	Qualitative, interviews	Not reported	Advanced practice clinicians	Three advanced practice clinicians
Kung et al.^60^/Mexico and Bolivia	Explores ‘the reasons for denial of legal abortion services in Mexico and Bolivia and identify ways to mitigate the misuse of conscientious objection’	Qualitative, in-depth interviews and focus groups	Professional contacts in each hospital identified eligible participants	Objecting and non-objecting OBGYN and generalists, allied health or physicians (not identified as objectors or non-objectors) in public hospitals	17 objecting and 17 non-objecting OBGYN and generalists 82 allied health or physicians (not identified as objectors or non-objectors)
La Roche et al.^61^/Canada	Aims to ‘document outcomes, identify facilitators and barriers, and distil learnings from an initiative that sought to recruit and support primary care clinicians in providing mifepristone/misoprostol in Canada’s capital’	Document analysis and in-depth interviews	Not reported	Clinicians who had received Medical Abortion Access Project training from five different sites	Clinicians who had received Medical Abortion Access Project training from five different sites
Lee et al.^62^/England and Wales	Explores the experiences of doctors who perform abortions	Qualitative, in-depth interviews	Not reported	Doctors who: had worked for a minimum of 10 years in abortion provision; who not only authorize but also perform abortions; abortion provision is the exclusive or major part of their work as Consultants in OBGYN or in Sexual and Reproductive Health	14 doctors who provide abortions
Marek^63^/USA	Examines ‘nurses’ attitudes toward pregnancy termination in the labor and delivery setting and the frequency of nurse refusal to care for patients undergoing pregnancy termination’	Survey with closed and open responses	Surveys distributed via Labour and Delivery Department managers	Labour and delivery nurses employed in central and Northern California hospitals	75 labour and delivery nurses
Martin et al.^64^/USA	Explores the tensions between abortion providers’ narratives and pro-choice discourse, and the types of narratives that are frequently silenced	Qualitative, analysis of Providers Share Workshop transcripts	Site-based liaisons recruited participants for the Providers Share workshop via emails, staff meetings, and flyers in staff areas	Abortion providers undertaking the Providers Share workshop at eight sites across the United States	96 abortion providers across a range of roles
Mavuso and Macleod^65^/South Africa	Explores how stigma may be resisted in social ways in pre-abortion healthcare encounters in the South African public health sector	Qualitative, narrative discourse approach, in-depth interviews	Not reported	Healthcare workers who participated in abortion across three hospitals where abortions were provided free of charge Patients who had undergone pre-abortion counselling	4 abortion providers (2 nurses, 2 counsellors) and 30 patients
Mavuso and Macleod^66^/South Africa	Explores experiences of pre-abortion counselling	Qualitative, constructionist narrative approach, semi-structured interviews	Not reported	Not reported	2 nurses employed at public hospitals 2 lay counsellors who volunteer for a Christian-based pregnancy crisis counselling NGO
Maxwell et al.^67^/Scotland and England	Explores ‘how health professionals may normalize abortion and challenge prevailing negative sociocultural Narratives’	Qualitative secondary analysis of interview data	Not applicable	Not applicable	Two qualitative datasets focusing on health professionals working in UK abortion provision
Mayers et al.^68^/South Africa	Explores the experiences of midwives who assist in abortions on a gynaecological ward	Qualitative, phenomenological, in-depth interviews	Not reported	Registered nurses and midwives actively participating in abortion provision on the gynaecological ward for 6 months or longer	3 midwives
McLean et al.^69^/Ethiopia	Explores abortion providers’ personal experiences, perceptions of the abortion law, and ethical dilemmas that arise	Qualitative, in-depth interviews and focus groups	Recruited from 4 public health centres, 2 hospitals, and 5 NGO clinics providing abortion plus other health services, for example, family planning, post-abortion care and other gynaecological services	Healthcare workers involved in any aspect of induced abortion including pre-abortion information and counselling, provision of MA or manual vacuum aspiration and post-abortion care	41 participants (including 24 nurses, 5 doctors, 3 health officers, 3 medical students, 1 pharmacist)
McLemore et al.^70^/USA	Describes ‘decision-making, using abortion as the clinical context to elucidate how nurses approach ethically challenging work’	Qualitative, in-depth interviews	Managers sent flyers to registered nurses and also posted flyers in staff-only areas in 14 San Francisco Bay area sites	Registered nurses who ‘could have encountered women needing abortions but for whom abortion was not the primary area of clinical expertise or practice’	25 nurses from the following range of settings: emergency departments, intensive care units, labour and delivery, post-anaesthesia care units and operating rooms
McLemore et al.^71^/USA	Explores ‘recruitment, retention and career development strategies for expert nurses in abortion care provision’	Qualitative, in-depth interviews	Managers sent flyers to registered nurses and also posted flyers in staff-only areas in 14 San Francisco Bay area sites	Nurses ‘who work (or have worked) with women seeking abortions in abortion clinics, emergency departments, labor and delivery units and post-anaesthesia care units’	16 nurses
McLeod et al.^72^/USA	Explores the influence of personal parenthood and pregnancy experiences on abortion providers	Qualitative, online anonymous qualitative survey	Sent survey to three electronic mailing lists, posted survey link in private Facebook groups for female abortion providers who are mothers, snowball sampling	Physicians who had provided abortions at any point in their careers	352 physician abortion providers
Mizuno^73^/Japan	Describes ‘midwives’ experiences in providing care in both pregnancy termination and Childbirth’	Qualitative, in-depth interviews	Informed all midwives in hospital of the study in writing plus verbally	Midwives with over 5 years of work experience in the delivery unit	11 midwives
Moller et al.^74^/Nepal	Explores experiences, views and attitudes of staff about working at safe abortion services in the Kathmandu Valley	Qualitative, in-depth interviews	Not reported	Doctors and nurses participating in induced abortion at 1 hospital and 5 clinics	15 abortion providers (including 11 doctors and 4 nurses)
Nicholson et al.^75^/England	Explores the experiences of gynaecological nurses involved in abortion	Interpretive Phenomenological Analysis, interviews and questionnaires	All eligible nurses within the service were invited to participate	‘Permanent, Registered Nurses, where part of their role was direct involvement with TOP’ in a public sector, ward-based TOP service	7 gynaecological nurses
O’Donnell et al.^76^/USA	Explores how the stigma of abortion provision can vary and strategies used to manage stigma	Qualitative, in-depth interviews	Purposive sample of members of a regional association of people involved in abortion care were contacted via email	Participants in a regional association involved in abortion care	7 physicians who perform abortions 2 physicians with abortion training who intend to perform abortions 2 certified nurse midwives who provide medication abortions, 2 registered nurses 1 social worker
Oppong-Darko et al.^77^/Ghana	Explores ‘midwives’ readiness for the provision of safe abortion services in Ghana’	Qualitative, in-depth interviews	Eligible midwives contacted via mobile phone number. Phone numbers were obtained from the District Health Directorate. Midwives were texted, and then phoned about participating. Midwives who were interested in participating were then sent information and a consent form.	Practising midwives in a district in Western Ghana	7 practising midwives
Ouedraogo and Juma^78^/Burkina Faso	Explores the views and experiences of patients and healthcare staff when seeking or delivering post-abortion care	Qualitative, observations in three referral-level health facilities and five primary-level health facilities and in-depth interviews	Not reported	Selected following observation of post-abortion care	13 abortion providers (including 2 obstetricians and gynaecologists, 9 midwives, 1 nurse, 1 licenced matron) Plus 39 women who sought post-abortion care
Parker et al.^79^/Canada	Explores ‘psychosocial, educational, and administrative support needs of labor and delivery (L&D) nurses who care for women undergoing pregnancy termination’	Qualitative descriptive design, semi-structured interviews	Convenience sample on L&D unit of a large metropolitan hospital. Clinical nurse specialist was a study champion, connecting potential participants with researchers	Full-time or part-time nurses on the L&D unit	10 nurses on the L&D unit
Pereira^80^/England and Wales	Explores abortion doctors’ accounts of their in the context of the medicalization of abortion	Qualitative, semi-structured interviews	Through closed forum for abortion providers, mailing list of Doctors for Choice, professional networks.	Doctors who provide abortion in England and Wales	47 doctors who provide abortion
Perrin et al.^81^/Switzerland	Explores healthcare professionals’ views on the changes to practice implemented post-legalization of TOP in French-speaking Switzerland	Qualitative, in-depth interviews	Heads of obstetrics and gynaecology departments assisted researchers with recruitment	Health professionals who worked in a service confronting TOP before and after legalization	77 healthcare professionals, including doctors, nurses and midwives, and sexual and reproductive health social workers
Persson et al.^82^/Bangladesh	Explores ‘health care providers’ perceptions and experiences of providing comprehensive abortion care in a humanitarian setting in Cox’s Bazar, Bangladesh and identifies barriers and facilitators in service provision’	Qualitative, in-depth interviews	Organizations that employed paramedics and doctors identified potentially eligible participants	Paramedic or doctor providing or supporting provision of menstrual regulation, post-abortion care and family planning, plus key informants (representatives of organizations supporting abortion care)	3 doctors, 16 paramedics and 5 key informants
Potgeiter and Andrews^16^/South Africa	Explores motivations and characteristics of nurses who become abortion providers	Qualitative, feminist, social constructionist approach, focus group plus individual interviews	Recruited via TOP workshop and state hospitals	22 abortion providers at state hospitals who had been nurses for 10 plus years	22 nurses who provided abortion in state hospitals
Purcell et al.^83^/Scotland	Explores the character, challenges and constraints of abortion provision	Qualitative, semi-structured interviews	Invited to opt-in in 2 hospitals and 1 sexual and reproductive health service	Health professionals involved in abortion provision	37 health professionals involved in abortion provision (including 17 nurses, 8 doctors, 7 clinical support workers and 5 sonographers)
Raifman et al.^84^/Tunisia	Explores ‘factors influencing provider attitudes about abortion and provider perspectives about abortion morality, safety, and legality’	Qualitative, in-depth interviews	Researchers contacted eligible participants via phone	Employed by a facility authorized to provide abortion Working with patients having abortions	7 physicians, 10 midwives, 2 nurses and 4 ‘gatekeepers’
Shaw^11^/Canada	Explores the experiences of physician abortion providers in Canada	Qualitative, narrative inquiry, in-depth interviews	Canadian Abortion Providers email list, presented about study at national meeting of abortion providers	Physician abortion providers	9 physician abortion providers, of whom 4 were the main participants for the study
Simmonds^85^/USA	Explores ‘the experiences of NPs [nurse practitioners] and CNMs [certified nurse midwives] who provide comprehensive early abortion care in New England’	Qualitative, descriptive study using individual interviews	Professional networks and snowballing	Nurse practitioners or certified nurse midwives currently providing abortion care	7 nurse practitioners and 1 certified nurse midwife/nurse practitioner
Teffo and Rispel^86^/South Africa	Explores coping strategies of abortion providers	Qualitative, in-depth interviews	Recruited from the public sector in two South African provinces	Doctors and nurses who had received formal abortion training and were providing legal abortions	30 nurse abortion providers
van Berkel^87^/Canada	Examines the experiences of abortion providers from hospital-based abortion services in Ontario	Qualitative, in-depth interviews	Contacted by letter, snowball sampling	Abortion providers within two hospital-based abortion services in Ontario	Eight abortion providers including medicine, nursing, social work, diagnostic imaging, and clerical roles
Wear^88^/USA	Explores ‘perspectives of physician abortion providers to understand more fully their motivations, the quality of their personal and professional lives, their views on the future of abortion services, and their recommendations for undergraduate and residency medical education’	Qualitative, in-depth interviews	Non-hospital providers contacted via databases including National Abortion Rights Action Leagues (NARAL), the National Abortion Federation (NAF) and the Alan Guttmacher Institute. Letters sent to providers at each location.	Physician abortion providers	7 physician abortion providers
Wolkomir and Powers^89^/USA	Explores ‘how one group of abortion clinic workers negotiated the difficulties associated with emotional labor in ways that allowed them to achieve this balance’	Qualitative, participant observation, loosely structured interviews	Clinic owner permitted researcher to work at the clinic and interview staff	Staff who had worked at the clinic for a minimum of 3 months. Doctors were excluded because they interacted minimally with the patients.	9 clinic workers (including 1 office manager, 5 counsellors, 1 counselling director, 1 receptionist and 1 back staffer)
Zwerling et al.^90^/USA	Explores ‘labor and delivery (L&D) nurses’ experiences caring for women undergoing induction for intrauterine fetal demise (IUFD) or termination for fetal anomalies’	Qualitative, semi-structured interviews	Eligible nurses were invited to participate via individual emails from the research team. Also, advertising flyers were situated in break rooms.	Nurses from a labour and delivery unit in a large metropolitan hospital	15 registered nurses

### Overview of the literature

The included studies were categorized into the key reasons that health providers participated in abortion.Table 3 shows the number of studies mapped to each category, as a proportion and percentage of the total number of included studies. The table demonstrates that the most cited reason for participating in abortion was to support women’s choices and advocate for women’s rights (76%). Other reasons included being professionally committed to participating in abortion (50%), aligning with personal, religious or moral values (39%), finding abortion provision satisfying and important (33%), being influenced by workplace exposure (19%), responding to community needs (14%), and participating for practical and lifestyle reasons (8%).

**Table 3.** Reasons for abortion participation, as a proportion and percentage of total included studies.

Category of reason	No. of studies/total no.	% of total studies
1. Supporting women’s choices and advocating for women’s rights	52/68	76
2. Being professionally committed to participating in abortion as health care	34/68	50
3. Aligning with personal, religious or moral values	27/68	39
4. Finding abortion provision satisfying and important	23/68	33
5. Being influenced by workplace exposure or support	13/68	19
6. Responding to community needs for abortion services	10/68	14
7. Participating for practical and lifestyle reasons	6/68	8

Each of these reasons will be explored below.

1. Supporting women’s choices and advocating for women’s rights.

Overall, 76% (52/68) of the included studies included supporting women’s choices and advocating for women’s rights as a reason for abortion participation.

Notably, 32 studies reported providers were motivated by a commitment to respecting women’s choices and their rights to self-determination and reproductive autonomy.^11,16,17,26–30,33,35–37,39,41,43–45,50,52,55,58,60,62,67,72,74,79,80,84,85,87,89^ This commitment extended to ensuring that women could have access to safe and lawful abortions. Present in most accounts was an explicit emphasis on the primacy of the woman as the decision-maker:

The bigger picture is of women in the world and individuals really in the world being able to make decisions about really personal things like reproduction.^62^ (p. 26)

In 27 studies, providers were motivated by a desire to protect women’s rights to health care and safe abortion.^11,16,28,29,31,35,38,40,42,46–49,52,53,56–58,60,66,69,74,77,78,80,82,88^ In some of these studies, providers were concerned about the high rates of morbidity and mortality from unsafe abortion particularly in vulnerable populations.^11,38,42,47,74,78^ For example, Fernandez Vazquez and Brown reported:

It became clear that, behind the maternal mortality and morbidity statistics, abortion was a social problem in which power played a part. The women suffering or dying in hospital emergency rooms were poor, uneducated, and young, among other vulnerabilities: ‘. . . they put their lives at risk only because of their social class situation, of poverty and of women. . .’.^47^ (p. 68)

In two studies, advocacy was motivated by a desire to correct historical injustices in medicine’s treatment of women.^11,26^ In Andaya and Campo-Englestein’s study about perceptions of pain and personhood in the periviable period, an abortion provider stated:

When I am taking care of an abortion, of a patient who is seeking an abortion, I am thinking exclusively about the woman. So when I am thinking about pain and what is acceptable pain, I am thinking about her pain . . . There is a lot of anti-woman and sort of misogynistic sentiment in my field for sure. And historically, obstetrics and gynecology was sort of built on women’s pain. So I have a very low bar for treating pain.^26^ (p. 4)

Around 15 studies reported that abortion providers perceived their work as a form of political activism or feminist advocacy.^11,16,17,29,31,35,41,44,45,47,64,80,85,87,88^

2. Being professionally committed to participating in abortion as health care.

Overall, 50% of the included studies (34/68) included professional commitments as a reason to participate in abortion care.

### Providing person-centred abortion care, even when abortion conflicted with personal values

In 23 studies, providers participated in abortion provision because of their duty to prioritize the welfare and well-being of their patients. This included providing health care to any patient in need, and without judgement.^17,26,28,30,32,34–37,40,48,51,52,54,58,60,70,73,74,78,85,88^ Providers also identified an obligation to uphold the Hippocratic Oath, comply with the law, adhere to health service protocols and standards, and the requirements of their individual role.

In eight studies, an important component of helping women in need was providing care that was not necessarily consistent with a provider’s personal or religious beliefs.^17,28,33,36,37,64,70,73,74^ For some providers, this meant separating their beliefs from their professional obligations. For example, in Czarnecki’s study, a participant stated:

Whoever ends up in front of me is my patient, and I owe them care . . . I can’t make decisions for other people. I can only make decisions for myself. Like I said before, [abortion] is not something that I could probably do myself. But the reason for participating is because I want to be a good care provider, and that’s understanding and nonjudgmental and it’s caring for whomever.^17^ (p. 184)

However, compartmentalizing personal or religious beliefs could sometimes be challenging, as identified by Martin:

However, other providers spoke about struggles with reconciling their work with messages about abortion from their churches. ‘I try to distance myself from that. . . I think my childhood growing up, 18 years of Catholic school, it’s still hard for me to accept what I do, even though I want to do this . . . and I’m fine with it. There’s still this inner struggle sometimes’.^64^ (p. 77)

### Abortion provision is within the scope of practice

In 15 studies, providers reported that abortion care was consistent with their professional scope of practice.^11,17,32,36,39,40,44,55,56,59,63,67,73,75,79^ In five studies, providers described abortion as a routine or normal part of health care more generally.^11,17,32,36,67^ Abortion was described variously as ‘just another surgery’,^32^ ‘a routine procedure’^36^ and ‘a normal part of women’s medicine’.^11^

### Abortion provision as comprehensive health care

In six studies, some providers were motivated to provide abortion services to ensure they offered comprehensive health services.^11,31,32,42,56,61^ Baird^31^ conducted an interview study with four Australian physician abortion providers and found that two of the providers moved into abortion provision as an extension of their medical practice in a related field. One participant was an obstetrician specializing in caring for women with serious medical issues, who wanted to give his patients ‘options’. Another was a doctor at a sexual health clinic who began providing medical abortions (MAs) after supporting a patient who had sourced her own methotrexate for an abortion.

3. Aligning with personal, religious or moral values.

In total, 39% of studies (27/68) included moral, religious or personal values as reasons for participating in abortion care.

### Personal beliefs and experiences

Around 12 studies included providers who were influenced to participate in abortion by their own beliefs and experiences, or the experiences of family and friends.^11,16,17,30,49,50,51,71,72,75,85,88^ These included histories of abortion, pregnancy (including unintended pregnancy), miscarriage, parenting (including raising children in low socioeconomic circumstances) and disability.

### Religious values

In 11 studies, providers reported they were motivated to participate in abortion care due to their religious beliefs (or perceived their religious beliefs were at least compatible with abortion provision).^16,17,28,46,49,56,64,69,77,86,90^ Providers drew on religious values, such as helping people in need, non-judgement, compassion and acceptance to justify their involvement in abortion care.^16,17,28,46,69,90^ A qualitative study of abortion providers in South Africa found a few providers perceived their participation in abortion was God’s will:

A few providers believed that termination of pregnancy provision was a calling from God. They reported that prayer gave them strength, and they coped by going to church, listening to gospel music and sharing with some church members who knew about the work the participant does at the hospital.^85^ (p. 345)

### Moral values

Moral values were evident in 11 studies where abortion providers described their involvement as a calling, passion or moral compulsion to serve.^11,32,35,38,41,56,65,67,69,70,86^ In a qualitative interview study of 31 physician and non-physician abortion providers in North Carolina working under restrictive abortion laws, the authors noted:

Overall, providers understood themselves to be performing altruistic work: ‘I felt that those patients really needed me and I felt, you know, it was necessary . . . Necessary and good and good work’ [sic].^35^ (p. 228)

Three studies included providers who were influenced to provide abortion because of their concerns for the quality of life of unwanted children.^56,75,81^ These concerns centred on the potential for children to be subjected to abuse and neglect:

I’m absolutely not against [TOP] [termination of pregnancy]. Personally I saw abused children, scalded, I saw babies in comas because they weren’t wanted. So you know, I think it’s better actually, to abort when it’s at the state of a comma, than an abused child. (45 NUR [nurse and midwife];^81^ p. 5)

4. Finding abortion provision satisfying and important.

Notably. 33% of studies (23/68) included the satisfaction and importance of abortion work as a reason for participation.

In 17 studies, providers reported they participated in abortion because of the significant and positive impact the procedure could have on a person’s life.^11,31,32,35,36,40,44,59,62,67,69,74,79,82,85,88,87^ The impacts of participating in an abortion were described variously. Sentiments included having ‘an enormous impact’ on the person’s future,^62^ providing a ‘big return on investment of [the providers] time’,^35^ ‘a watershed experience in [pregnant people’s] lives’,^36^ ‘alter[ing] the course of a woman’s life’,^59^ ‘relieving a woman of her burden’,^74^ ‘an existential experience’^11^ and ‘rescu[ing] someone from a miserable life in a matter of a few hours’.^88^

In 11 studies, abortion providers described feeling satisfied that they were able to provide the care women needed.^30–32,34,54,74,78,80,83,85,88^ Some providers reported being motivated to continue provision due to the gratitude expressed by their patients:

Midwives understand the grief and sorrow women, their partners and families suffer during TOP [termination of pregnancy]. They are passionate about supporting women’s choices, facilitating a positive birthing experience and helping women become mothers within the space of losing their babies. Knowing, through the joy of receiving a written note or a word of gratitude, that women are satisfied with their care is immensely rewarding for midwives. It is their incentive to do it all over again.^30^ (p. 621)

In one study, providers were inspired by their perception that the broader community valued their work:

Participants also described broad support for their role providing abortion outside of work, including from their partners, friends, family and neighbors. This seemed to contribute to the general feeling that providing this type of care was a positive experience.^85^ (p. 65)

5. Being influenced by workplace exposure or support.

Overall, 19% of studies (13/68) were mapped to this category.

In 13 studies, providers reported they were influenced by their workplace to participate in abortion care.^11,16,17,29,30,34,65,66,68,71,80,82,85^ Sometimes, the influence stemmed from organizational cultures where abortion provision was positively regarded and actively supported, as evidenced by the investment and availability of abortion training and mentoring opportunities. In addition to organizational support, some providers were motivated by supportive colleagues:

Across both of these groups, four participants relayed that a specific person had been particularly important in inspiring or mentoring them to become a provider of abortion care. For Sandy, this was the nurse who supervised her in her college work-study position. As she described her, ‘Adele was the muse, the mentor, the person who got me from a high school kid from [name of city where she grew up] to what I do today, by showing me the importance of women’s health care’.^85^ (p. 78)

Studies have reported some providers participated in abortions because they were asked to, or because provision was a requirement of their role. Exposure to abortion in previous workplace settings, including in other countries, also served as a reason:

The other key influence to emerge was exposure of health providers to abortion-care provision in other settings. Many obstetrician-gynaecologists have worked, trained or travelled abroad professionally and their attitudes were striking: ‘I worked in the UK for several years and I offered terminations and you are not paid for it, but it is just a service you are providing and because you believe in it that if you don’t do that maybe something worse will happen’.^29^ (p. 8)

6. Responding to community needs for abortion services.

Around 14% of studies (10/68) included responding to community needs as a reason for abortion participation. The studies reported that some providers felt an obligation to provide abortion services, due to a dearth of other providers.^11,17,32,38,39,44,63,65,66,88^ In these situations, providers participated in abortion care due to concerns about negative ramifications if they did not:

For others, the availability of providers in the region shaped their participation decisions. Another MFM [maternal-fetal medicine] specialist described how a colleague held similar beliefs about abortion, but the contexts in which they worked led them to very different participation decisions:

‘When [my colleague] trained, no one else did terminations. And she said, “Well, if anyone’s gonna have access to this, I need to learn how to do it and offer this”. [We both feel] as professionals that we need to offer women uniform service, but since she was surrounded by people who refused to perform it, she ended up doing terminations. And I was surrounded by lots of people who did terminations, so for me it was easier to not do terminations. So . . . the same values and the same goals result in very opposite decisions just based on the circumstances that we were in’.^17^ (p. 185)

7. Participating for practical and lifestyle reasons.

Overall, 8% of studies (6/68) were mapped to this category.

Five studies included providers who chose abortion provision for pragmatic reasons. These reasons included that the job provided an income or better work-life balance.^32,64,69,80,89^ Only one study indicated that abortion provision could be profitable:

Financial motivations were also evident in some of the pharmacy workers’ descriptions of their gatekeeping decisions, as some made clear that ‘of course on the personal interest, again, there is money’ and considered the product a profitable medication. However, most only mentioned the need to prevent unsafe abortion when asked about the benefits of selling MA [medical abortion].^48^ (p. 188)

## Discussion

This scoping review identified a range of reasons which contributed to health providers’ decisions to participate in abortion care. Studies demonstrated that abortion provision was consistent with health providers’ professional obligations to provide person-centred care, to work within their scope of practice and to provide services that were responsive to community and patient needs. These are core obligations of mainstream health providers. The review does not support public portrayals of abortion providers as illegitimate, dangerous or greedy. Indeed, in contrast to images portraying abortion providers as dangerous and negligent, many of the included studies demonstrated providers wanted to support women’s choices and advocate for their rights to safe abortion services. Moreover, representations of abortion providers as greedy were not supported by our findings. Instead, we found many studies that showed abortion providers were motivated by moral or religious values, including altruism. Only one of the 68 studies showed that profit played a role for some pharmacists in the stocking of abortion medication.

The findings of this review also challenge attitudes that abortion provision is an exceptional, rather than routine, part of health care.^91^ Singling out abortion, without empirical justification, from other parts of medicine reinforces abortion stigma.^92^ Being passionate, wanting to help people in need and being engaged in satisfying work are not motivations unique to abortion providers. For example, Omar^93^ found medical students in Malaysia were motivated to pursue medicine by passion and interest, and the desire to help. In another study, Newton et al.^94^ demonstrated that nurses and nursing students in Australia chose their profession because of a desire to help, a sense of achievement and self-validation. By providing evidence that abortion providers share key motivations with health professionals more broadly, this review could contribute to normalizing abortion provision as routine health care.

The findings of this review support previous research that health providers can be motivated to participate in abortion by their deeply held, core values.^10^ These values included non-judgement, compassion and altruism. This finding supports arguments that conscientious provision of abortion should be recognized.^8,9,95,96^ Given that conscience clauses aim to protect moral integrity, and a clinician’s moral integrity may be harmed through not being able to provide abortion (for example, due to institutional or legislative restrictions), then a lack of protection for positive claims of conscience may be unjustified.^9^ In jurisdictions where abortion is lawful but banned by individual institutions, Fox^97^ argues that the grounds for protecting conscientious provision are stronger when there are not enough clinicians in nearby facilities to provide the procedure. The case for accommodation is also strong when the additional costs for the institution are minimal. For instance, allowing a practitioner to prescribe MA does not incur the same resourcing costs (e.g. staff and equipment) as surgical abortions. While accommodating conscientious providers may raise complicity concerns for objecting institutions, there are strategies that institutions can employ to distance themselves to minimize these. For instance, permitting a conscientious provider to prescribe MA via telehealth or, as Fox suggests, consigning abortion provision to an off-site facility may mitigate complicity to some extent. However, like protecting conscientious refusals, protecting positive claims of conscience may not be straightforward in practice. This is particularly so in cases where abortion is not lawful in a jurisdiction; in such cases, accommodation of conscientious provision may not be possible.

In addition to conscience-based decision-making, the findings demonstrated other similarities between the reasons of providers and those who refuse to participate. Both provision and refusal can be motivated by professional ethics, including the Hippocratic Oath.^15^ Workplace experiences may also motivate both provision and refusal. A key difference may be that those who refuse to participate can be influenced by emotional labour considerations, such as fear of the emotional aspects of care provision and concern about stigma and judgement.^15^ The findings of this review, however, suggest that some health practitioners who provide abortion, including providers who are personally opposed to abortion, negotiate emotional labour considerations without refusing care. Previous research has identified that factors including positive feelings about abortion work and team support can help to sustain abortion care participation.^98,99^ However, further research comparing how providers and those who refuse to provide manage emotional labour considerations is warranted and could provide further insights.

### Strengths and limitations

This scoping review included a large volume of studies from a range of geographic regions, types of abortion providers and health service settings. The rigour of the review was strengthened by a protocol, dual screening of titles, abstracts and full-text articles, and comprehensive academic database and grey literature searches.^18,19^ Consistent with a scoping review, no critical appraisal of studies was undertaken, and the certainty of findings was not rated.^19^

This review has several limitations. First, due to the limited number of studies solely focused on abortion providers’ reasons, many of the studies included in this review were indirectly related to our research question. This meant that although they included relevant data about reasons for provision, this was not their specific focus. Second, to determine whether studies occurred in jurisdictions where abortion was lawful, we relied on individual study authors’ descriptions of the abortion law, rather than confirming the lawfulness of abortion in that jurisdiction independently. Finally, while the broad definition of ‘health provider’ captured a wide range of studies, it is possible that some reasons may be more relevant for some types of providers than others. For example, narrowing the population to providers engaged in direct provision may have yielded fewer, but more relevant, reasons for that population.

More in-depth analysis of reasons could be explored in future qualitative evidence syntheses of specific subgroups. These could include potential differences in provider reasons according to the type of abortion (e.g. medical or surgical), length of gestation (e.g. abortion in the first trimester versus abortion in second or third trimesters) or reason for abortion (e.g. foetal anomalies, rape, maternal interests, etc.). Such analyses were not possible within the broad remit of a scoping review.

## Conclusion

This scoping review demonstrated that health providers who participated in abortion provision were motivated by a range of reasons, including support for women’s choices, professional commitments, personal, moral and religious beliefs, the satisfying nature of abortion work and exposure to abortion in the workplace. The findings did not support the negative portrayals of abortion providers that exist in public discourse. When compared with research about reasons for conscientious objection, the review also showed similar factors can motivate both participation and non-participation (e.g. religious or moral beliefs, professional commitments and workplace experiences). The nature of these reasons may help to challenge the discourse that conscience is associated solely with objection to abortion. Efforts to recognize conscience-based provision could be explored.

## Supplemental Material

sj-docx-1-whe-10.1177_17455057241233124 – Supplemental material for Health providers’ reasons for participating in abortion care: A scoping review Supplemental material, sj-docx-1-whe-10.1177_17455057241233124 for Health providers’ reasons for participating in abortion care: A scoping review by Bronwen Merner, Casey M Haining, Lindy Willmott, Julian Savulescu and Louise A Keogh in Women’s Health

sj-docx-2-whe-10.1177_17455057241233124 – Supplemental material for Health providers’ reasons for participating in abortion care: A scoping review Supplemental material, sj-docx-2-whe-10.1177_17455057241233124 for Health providers’ reasons for participating in abortion care: A scoping review by Bronwen Merner, Casey M Haining, Lindy Willmott, Julian Savulescu and Louise A Keogh in Women’s Health

## Declarations

**Ethical approval and consent to participate:** Ethical approval and consent to participate is not applicable because all data were from already published materials.

**Consent for publication:** Not applicable

**Availability of data and materials:** The protocol for this scoping review was published on the Open Science Framework on 13 January 2022 at the following doi: https://osf.io/yhsr5. All data and materials in the included studies are available open access or via subscription to the following academic databases: MEDLINE (Ovid), Embase (Ovid), CINAHL (EBSCO), ScienceDirect and CAB Abstracts (including Global Health).
