# Supplementary material for: Health providers’ reasons for participating in abortion care: A scoping review
Source: Womens Health (Lond). 2024 Mar 1;20:17455057241233124. doi: 10.1177/17455057241233124 (PMC10908244; doi:10.1177/17455057241233124)
Supplement: sj-docx-2-whe-10.1177_17455057241233124 – Supplemental material for Health providers’ reasons for participating in abortion care: A scoping review [file sj-docx-2-whe-10.1177_17455057241233124.docx]

**Appendix 1: Search strategies**

**Medline search strategy**

1 exp Physicians/ 172541

2 exp Gynecology/ 20472

3 exp Obstetrics/ 24349

4 Nurses/ 44779

5 exp Midwifery/ 20989

6 Medical Staff/ or Nursing Staff, Hospital/ or Nursing Staff/ or Medical Staff, Hospital/ 92789

7 Allied Health Personnel/ 12881

8 (provider* or staff or worker*).mp. [mp=title, book title, abstract, original title, name of substance word, subject heading word, floating sub-heading word, keyword heading word, organism supplementary concept word, protocol supplementary concept word, rare disease supplementary concept word, unique identifier, synonyms] 685158

9 1 or 2 or 3 or 4 or 5 or 6 or 7 or 8 928796

10 Abortion, Induced/ or Abortion, Legal/ 36043

11 ("termination of pregnancy" or "interruption of pregnancy").mp. [mp=title, book title, abstract, original title, name of substance word, subject heading word, floating sub-heading word, keyword heading word, organism supplementary concept word, protocol supplementary concept word, rare disease supplementary concept word, unique identifier, synonyms] 7443

12 10 or 11 40463

13 9 and 12 4266

14 (motivat* or reason* or factor* or influen* or commit* or inspir* or identity or voice* or experien*).mp. [mp=title, book title, abstract, original title, name of substance word, subject heading word, floating sub-heading word, keyword heading word, organism supplementary concept word, protocol supplementary concept word, rare disease supplementary concept word, unique identifier, synonyms] 9417137

15 13 and 14 1995

16 limit 15 to (english language and humans and yr="2000 -Current") 1140

**CINAHL**

|  |  |  |  |
| --- | --- | --- | --- |
| \| S21 \| S18 AND S19 \| Limiters - Published Date: 20000101-20220231; Exclude MEDLINE records; Language: English Search modes - Boolean/Phrase \| 682 \| \| --- \| --- \| --- \| --- \| \| S20 \| S18 AND S19 \| Search modes - Boolean/Phrase \| 1,933 \| \| S19 \| motivat* OR reason* OR factor* OR influen* OR commit* OR inspir* OR identity OR voice* OR experien* \| Search modes - Boolean/Phrase \| 2,554,357 \| \| S18 \| S10 AND S17 \| Search modes - Boolean/Phrase \| 3,674 \| \| S17 \| S11 OR S12 OR S13 OR S14 OR S15 OR S16 \| Search modes - Boolean/Phrase \| 24,314 \| \| S16 \| "interruption of pregnancy" \| Search modes - Boolean/Phrase \| 32 \| \| S15 \| "termination of pregnancy" \| Search modes - Boolean/Phrase \| 1,830 \| \| S14 \| abortion \| Search modes - Boolean/Phrase \| 23,322 \| \| S13 \| (MH "Abortion, Habitual") \| Search modes - Boolean/Phrase \| 918 \| \| S12 \| (MH "Abortion, Incomplete") \| Search modes - Boolean/Phrase \| 186 \| \| S11 \| (MH "Abortion, Induced+") \| Search modes - Boolean/Phrase \| 12,323 \| \| S10 \| S1 OR S2 OR S3 OR S4 OR S5 OR S6 OR S7 OR S8 OR S9 \| Search modes - Boolean/Phrase \| 1,344,621 \| \| S9 \| workers or employees or staff or personnel \| Search modes - Boolean/Phrase \| 529,980 \| \| S8 \| (MH "Multidisciplinary Care Team") \| Search modes - Boolean/Phrase \| 50,544 \| \| S7 \| (MH "Personnel, Health Facility+") \| Search modes - Boolean/Phrase \| 44,319 \| \| S6 \| (MH "Social Workers") \| Search modes - Boolean/Phrase \| 10,867 \| \| S5 \| (MH "Health Personnel+") \| Search modes - Boolean/Phrase \| 631,422 \| \| S4 \| (MH "Gynecology") \| Search modes - Boolean/Phrase \| 5,144 \| \| S3 \| (MH "Obstetrics") \| Search modes - Boolean/Phrase \| 6,955 \| \| S2 \| (MH "Nurses+") OR (MH "Midwives+") OR nurse OR midwives \| Search modes - Boolean/Phrase \| 640,392 \| \| S1 \| (MH "Physicians+") or doctors \| Search modes - Boolean/Phrase \| 185,246 \| |  |  |  |

**CAB Abstracts**

| S10 | S8 AND S9 | Search modes - Boolean/Phrase | 563 |
| --- | --- | --- | --- |
| S9 |  | Limiters - Publication Year: 20000101-20220131 Search modes - Boolean/Phrase | 7,257,183 |
| S8 | S5 AND S6 AND S7 | Search modes - Boolean/Phrase | 650 |
| S7 | (S1 OR S2 OR S3 OR S4) | Search modes - Boolean/Phrase | 135,925 |
| S6 | AB experiences OR perceptions OR motivations | Search modes - Boolean/Phrase | 264,600 |
| S5 | AB (provider* OR worker* OR staff OR nurse* OR doctor* OR obstetric* OR physician* OR midwi* OR "social worker") | Search modes - Boolean/Phrase | 237,567 |
| S4 | AB top | Search modes - Boolean/Phrase | 107,001 |
| S3 | AB "interruption of pregnancy" | Search modes - Boolean/Phrase | 34 |
| S2 | AB termination | Search modes - Boolean/Phrase | 12,019 |
| S1 | SU abortion | Search modes - Boolean/Phrase | 17,509 |

**ScienceDirect**

Year: 2000-2022 Title, abstract, keywords: (abortion AND (providers OR nurses OR doctors OR physicians OR obgyn OR obstetric) AND experiences OR perceptions

Number of search results: 1,426

**EMBASE**

11 (provider* or professional* or staff or worker* or nurs* or midwi* or "social work*" or "maternal fetal medicine" or "obstetrics and gyn*" or obgyn or physician* or doctor*).mp. 2938779

12 (abortion* or termination* or TOP or "interruption of pregnancy").mp. 443982

13 (motivat* or reason* or factor* or influen* or commit* or inspir* or identity or voice* or experien*).mp. 11255225

14 (qualitative or mixed method*).mp. 454949

15 11 and 12 47749

16 13 and 15 23197

17 14 and 16 2299

18 limit 17 to humans 2211

19 limit 18 to english language 2177

20 limit 19 to exclude medline journals 157

21 limit 20 to yr="2000 -Current" 69

**Appendix 2: Differences between protocol and review**

The following changes to the selection criteria were made between the protocol and the review:

- The protocol included qualitative studies only. In the review, we included quantitative designs as well to ensure a comprehensive overview of the literature.
- In the protocol, we did not clarify whether student health providers would be included. In the review, students were included because their experiences were considered relevant to provision once qualified.
- In the review, we found a high number of studies about education and training interventions (e.g. participation in abortion training). These were excluded because many of the papers did not directly relate to providers’ reasons.
